# Dynamic Navigation for Zygomatic Implants: A Case Report about a Protocol with Intraoral Anchored Reference Tool and an Up-To-Date Review of the Available Protocols

**DOI:** 10.3390/mps3040075

**Published:** 2020-11-05

**Authors:** Gerardo Pellegrino, Giuseppe Lizio, Francesco Basile, Luigi Vito Stefanelli, Claudio Marchetti, Pietro Felice

**Affiliations:** 1 Oral of Maxillofacial Surgery Unit, Department of Biomedical and Neuromotor Sciences, University of Bologna, Via Massarenti 9, 40138 Bologna, Italy; giuseppelizio@libero.it (G.L.); basile@dottorbasile.it (F.B.); claudio.marchetti@unibo.it (C.M.); 2 Department of Oral and Maxillo-Facial Sciences, Sapienza University of Rome, 00185 Rome, Italy; gigistef@libero.it; 3 Oral Surgery Unit, Department of Biomedical and Neuromotor Sciences, University of Bologna, Via Massarenti 9, 40138 Bologna, Italy; pietro.felice@unibo.it

**Keywords:** dynamic navigation, image-guided surgery, reference tool, zygomatic implants, fiducial markers

## Abstract

Dynamic Navigation is a computer-aided technology that allows the surgeon to track the grip instruments while preparing the implant site in real time based on radiological anatomy and accurate pre-operative planning. The support of this technology to the zygoma implant placement aims to reduce the risks and the errors associated with this complex surgical and prosthetic treatment. Various navigation systems are available to clinicians currently, distinguished by handling, reliability, and the associated economic and biological benefits and disadvantages. The present paper reports on the different protocols of dynamic navigations following a standard workflow in correlation with zygomatic implant supported rehabilitations and describes a case of maxillary atrophy successfully resolved with this technology. An innovative and minimally invasive dynamic navigation system, with the use of an intraoral anchored trust marker plate and a patient reference tool, has been adopted to support the accurate insertion of four zygomatic implants, which rapidly resolved maxillary atrophy from a 75-year-old male system. This approach provided an optimal implant placement accuracy reducing surgical invasiveness.

## 1. Introduction

Pre-implant bone reconstructions can now often be avoided thanks to the introduction of short, narrow, and standard-length tilted implant [1,2,3]. In cases of severe atrophy of the jaw-bones, reconstructive techniques before implantology demonstrated several limits; grafting or tissue engineering procedures, with the use of growth factors and contenting devises, do not often obtain the hoped results due to the compromised vascularization and the anatomy of the residual alveolar ridge. In addition, these procedures resulted lengthy and demanding [4]. Zygomatic implants (ZIs) are used for the rehabilitation of severe maxillary atrophies and defects with a >95% survival rate after nearly 13 years of follow-up [5,6,7]. This approach, with shorter treatment times, the possibility of an immediate loading, and reduced surgical morbidity, is considered difficult and risky due to the complexity of the zygomatic bone in atrophic or resected patients, the limited direct visibility and the long pathway of implant site preparation [8]. Several important surgical complications, such as sinusitis (5.86%), peri-implant mucositis (2.96%), nerve injuries (1.26%), oroantral fistula (1.20%), or even orbital perforation or infratemporal fossa invasion (1.33%) have been reported [5,9]. Furthermore, a good connection with the prosthetic structure strongly depends on the correct positions of the fixtures, with the risk of a too palatal implant emergence, a wrong distribution of masticatory forces, and implant mobility and prosthetic fractures [5,10,11,12,13]

A pre-operative plan of the correct position of ZIs with dedicated software, according to the anatomical and prosthetic scenario, must be followed by a precise system to transfer the virtual project to the clinical reality. The printed surgical template in a computer-guided (static) system is not able to direct the burs and the fixtures to the right position due to the length of the drill path-way, with a considerable deviation of the planned axes [8,14,15,16,17]. A navigation-guided (dynamic) system allows to continuously track the position of the drills and implants in real time by superimposing them on the radiological anatomy and the pre-operative planned position [8,18]. The dynamic navigation (DN) was borrowed from neuro- and maxillo-facial surgery and was gradually adapted to implantology, reducing the bulkiness of structural components, and maintaining a high level of correspondence between the virtual and the real domain. Two main aspects can differentiate and influence the DN systems quality: the fiducial markers number and position and the anchorage and stability of the patient reference tool. The first point influences the registration/calibration process that links the virtual and the patient coordinate systems; problems in this phase can affect the predictability of the entire procedure. The second point conditions the tracking of the handle instruments in relation to the patient position detected by a camera. The evolution from an invasive cranial anchorage used in major surgeries [18] to more manageable intra-orally connected patient reference tool [10,19,20] made the DN supported implantology less invasive and more ergonomic.

Since Schramm et al. [21] first successfully inserted a ZI supported by DN technology, in vitro experiments and case reports have testified the usefulness of this procedure in terms of predictability, shortening treatment times, and morbidity reduction as compared to a free-hand approach [20,22,23,24]. Most of the studies about DN supported ZIs placement employed bulky system with cranial anchorage patient reference tools [8,20,22,24]. Lopes et al. [20] reported a case treated with an intra-oral reference tool anchorage, while Pellegrino et al. adopted a DN technology with intra-oral anchorage and no invasive fiducial markers in partially resected maxillae [25] and with an ultrasonic implant path way preparation [10]. The use of ultrasonic instruments further reduces the invasiveness in implantology [26].

We report a case of extreme maxilla atrophy treated with ZIs insertion supported by DN system; this is characterized by a unique minimally invasive frame carrying both fiducial markers and an intraoral patient reference tool. A workflow of the DN protocols taking into account the technology currently available is illustrated with comments and discussions.

## 2. Case Report

**Figure 1:**
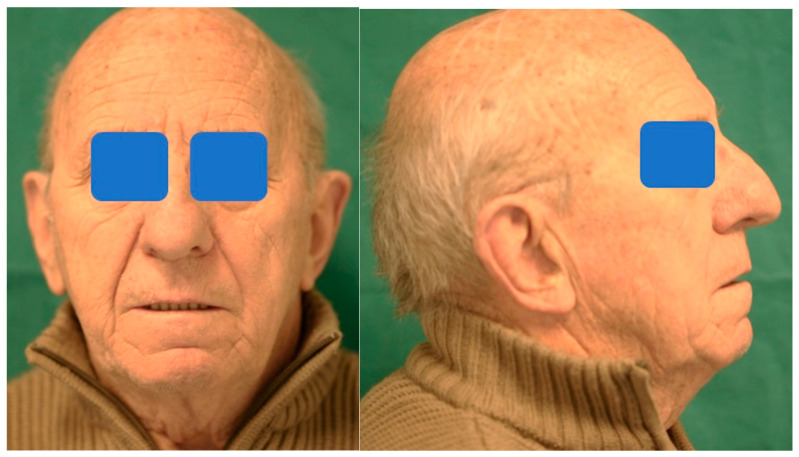
Pre-operative frontal and lateral view of the patient.

A 75-year-old man was referred to the Oral and Maxillofacial Surgery Unit of the Department of Biomedical and Neuromotor Science, University of Bologna. The patient was in good general health and he was wearing a removable maxillary prosthesis and a mandibular implant-supported overdenture. The primary complaint of the patient was to restore the instability of the upper denture, adversely affecting speech and masticatory function, with a fixed denture. Following the clinical and radiological examination, a horizontal bone atrophy was evidenced, at the level of the premaxilla, and severe bone resorption posteriorly (Figure 1).

**Figure 2:**
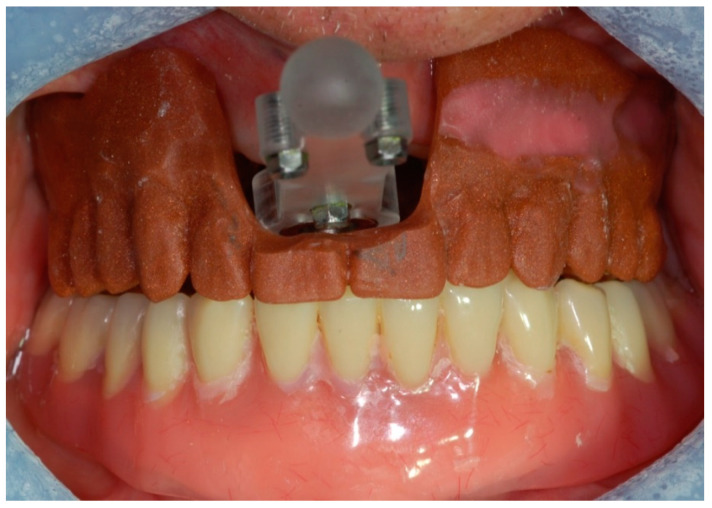
The markers plate and the radiographic template placed in situ for the CBCT.

The patient refused any bone grafting and required the quickest possible solution. Several treatment options were evaluated: narrow, tilted, pterygoid, intra-sinus, and zygomatic implants. The patient opted for a zygomatic implant rehabilitation. Consequently, upon completion of the informed consent form, the patient was enrolled in a clinical trial approved by the Ethical Committee of S. Orsola-Malpighi University Hospital, Bologna, Italy. A provisional denture was made to investigate the inter-arch relationship and occlusion for planning an immediate loading. A resin-based duplicate of the prosthesis was prepared as a radiographic template. A provisional narrow implant with the coronal spires machined (MSC-IBNT, Southern Implants, Irene, South Africa) was placed under local anesthesia with a flapless technique into the premaxilla. A marker plate containing the fiducial markers for the navigation system calibration was directly screwed onto the provisional implant. After removing any interference, the radiographic template was placed in situ (Figure 2) and the Cone-Beam Computed Tomography (CBCT) was taken.

**Figure 3:**
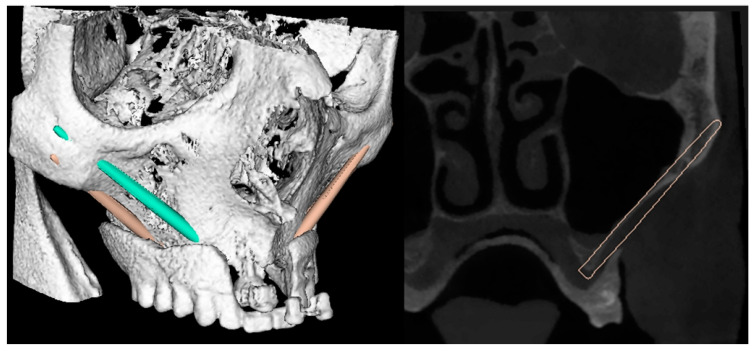
The three-dimensional implant planning and the cross-sectional view of the implant trajectory planned according to the prosthesis.

Then, the marker plate was unscrewed and repositioned on the day of the surgery. The ImplaNav dynamic navigation system (BresMedical, Sydney, Australia) was used for zygomatic implant planning and placement. The planning of the ZIs position was made using the navigation system software. The three-dimensional implant trajectory was planned according to the prosthesis position aiming to reach a good prosthetic emergence (Figure 3).

**Figure 4:**
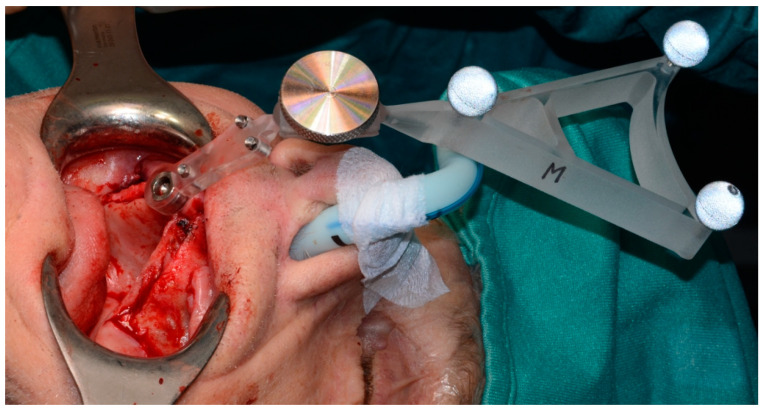
The intraoral reference tool in situ.

The intervention was carried out under general anesthesia and with local infiltration. Before the surgery, the two reference tools, both consisting of a support for three reflective spheres (required by the navigation system camera for the real-time tracking) but with different geometries, were fixed: one onto the patient directly jointed on a spherical connection on the marker plate, and the second onto the surgical handpiece (Surgysonic^®^ Moto, Esacrom, Imola, Italy) (Figure 4). 

**Figure 5:**
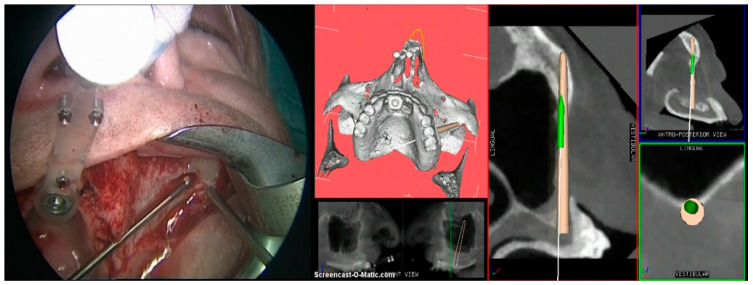
The preparation of the zygomatic implant site following the drill tracking and the planned implant trajectory on the screen of the navigation system (right side) in real time.

In this way, the infrared camera of the navigation system could record both the position of the patient and the surgical handpiece and the system could display onto the screen the drill position on the patient’s CBCT images. The system registration/calibration proceeded with the use of the three fiducial markers. They are located on the marker plate and must be touched in sequence with the first drill to allow the navigation system to identify the patient position. The system can detect the position of the handpiece by setting up the calibration tool as a drill and exposing it for a few seconds at the navigation system camera. A full-thickness flap enables access and visualization of the malar bone. The site preparation for the zygomatic implant, from the residual alveolar ridge to the zygomatic bone, was done using the navigation system that tracked each drill. The surgeon was able to follow the intended trajectory of the implant on the real-time navigation system screen (Figure 5).

**Figure 6:**
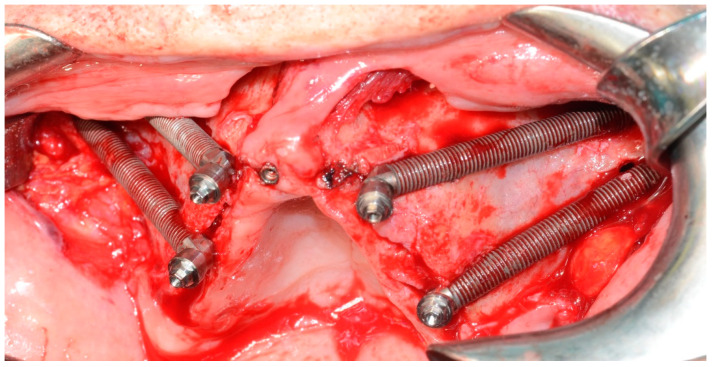
Four zygomatic implants and four conical abutments positioned before the sutures.

An extra-sinus technique was used due to the favorable anatomy of the patient. In total, four ZIs (Southern Implants, Irene, South Africa) were placed to achieve a fixed full-arch implant support. The provisional implant was considered stable and integrated, and it was used for the provisional prosthesis support. The prosthesis was manufactured, allowing the provisional implant to be removed in the future if required. Four conical abutments were positioned before suturing (Figure 6). 

**Figure 7:**
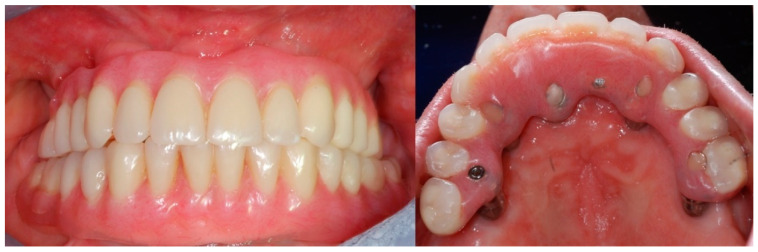
The screw-retained immediate loaded prosthesis and the occlusal view of the implant prosthetic emergences.

Four pick-up transfers were then placed and rigidly connected with a hard resin (Duralay, Reliance, Worth, USA) and impressions were taken using a customized tray and polyether impression material (Impregum; 3M ESPE, Seefeld, Germany). Within 72 h, a screw-retained metal-resin prosthesis was delivered and connected to ZIs (Figure 7). 

**Figure 8:**
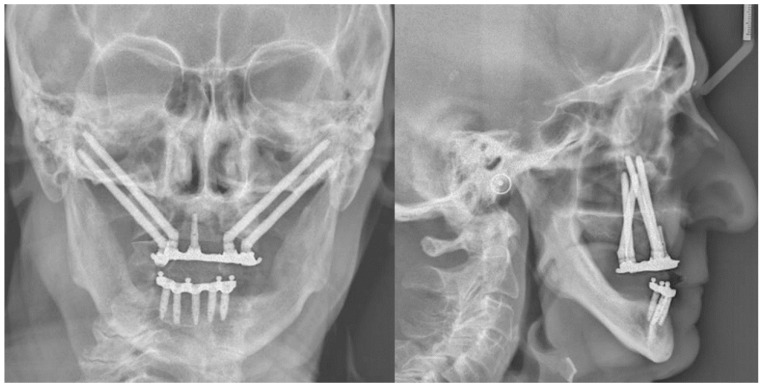
Postoperative frontal and lateral radiographs.

**Figure 9:**
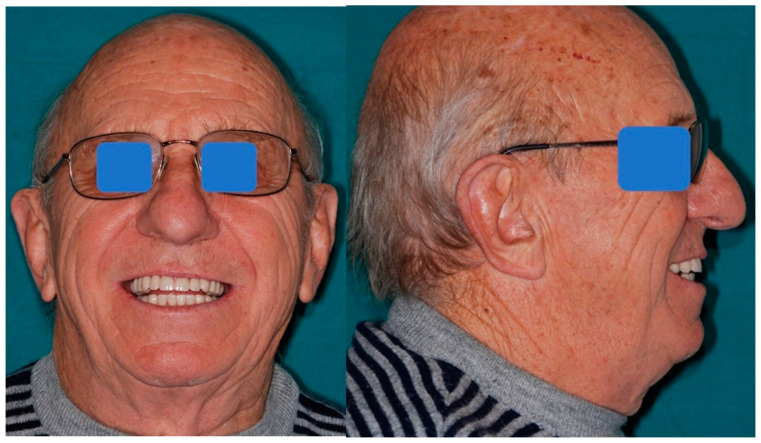
Postoperative frontal and lateral view of the patient.

Postoperative radiographs were performed and sutures (Vicryl, Somerville, MA, USA) were removed after 2 weeks. Clinical and radiological follow-ups were performed at 1, 3, 6, and 12 months after implant placement showing good soft and hard tissue conditions. No intraoperative nor postoperative complications occurred (Figure 8 and Figure 9). 

## 3. Procedure

1. Step 1. Scanning of the patient and the scan prosthesis/registration template, external registration frame, or bone markers.
2. Step 2. Software planning of the implant position.
3. Step 3. Image-to-patient registration via registration templates, external registration frames, or bone markers.
4. Step 4. Surgery: navigation of the drill along the predefined surgical plan.

## 4. Discussions

Zygomatic implantology can benefit from DN support technology more than static computed guidance. Since the drill path is about four times longer than that for standard fixtures, the stereolithographic templates used in static computer aided systems adds small angular or positional entrance errors, resulting in a risky apical tip moving away from the planned safe position. Hung et al. [23] reported an entry deviation, exit deviation, and angle deviation of 1.35 ± 0.75 mm, 2.15 ± 0.95 mm, and 2.05° ± 1.02°, respectively, for DN-supported ZIs placement. These values are more acceptable than the entry and exit deviation of 2.77 and 4.46 mm, respectively [15], and the angle deviation of 8.06° in antero-posterior view and 11.20° in caudal-cranial view [14] in static computed guided technology. The values of Hung et al. are comparable to those reported for navigated standard implants and confirm a good accuracy of this technique [10,22,27,28,29]. Moreover, differently from static computed systems, DN allows the clinician to modify the surgical plan as the clinical situation requires, which is quite useful without pre-operative information about the real bone structure and important for the level of primary stability that ZIs are supposed to obtain [18].

The reliability of DN technology depends on systematic and non-systematic errors. The first category is linked up to the inherent starting characteristic of the used instrumentation, while the second depends on a non-appropriate use of the machine for operator inexperience or for setbacks. Systematic errors can be categorized in relation to the different types of technologies that may impact tracking or registration processes. The CT/CBCT image characteristics influence both tracking and registration phases. The set thickness of the CT layer at 0.625 to 1.0 mm and the voxels size from 0.3 to 0.5 mm are considered good for accuracy in ZIs placement procedure [8].

Tracking is related to the dynamically continuous following of the surgical instruments in the space in relation to patient position and the previous acquired radiological anatomy and implant insertion planning. Two principal tracking systems are available so far: optical and electro-magnetic [30]. Most of the dynamic navigators used in surgical dentistry are optical-based, distinguished in active and passive: in active-tracking, a battery-powered light is enclosed in bulky reference tools emitting luminous spots to be detected by two infrared cameras; in passive-tracking, one camera produces infrared flashes, illuminating the reference tools consisting of light-reflecting spheres, with the necessity for operators not to spatially interfere between the spheres and the camera [31]. 

Registration is the determination of the relationship between the imaging data and the surgical field, allowing the system to associate any point on the patient to the correspondent imaging datum [8,32]. The reliability of DN in terms of precision crucially depends on this phase. The “paired-point” registration is the most diffuse procedure and depends on the presence of fiducial markers (FMs) to be recognized by a probing tool both on the patient and the virtual imaging. The positions and numbers of FMs influence all the procedure in terms of potential location errors. These errors can derive from fiducial localization error (FLE), fiducial registration error (FRE), and the target registration error (TRE): the last is the most reliable parameter because related to the correspondence between the surgical tool and the surgical target coordinates [8]. The method of using bone-anchored screws as FMs is considered as the gold standard of “paired-point” registration [33]. The mini screws are inserted in the maxillary bone involving the nasal spine, the canine fossa, the maxillary tuberosity, and the mid-palatal suture, before taking the cone beam CT. According to Fan et al. the minimal number of FMs to have an acceptable target registration error (TRE) is five, placed in a polygonal distribution; in case of fewer than six FMs, a regular triangle distribution, with the base in correspondence of the posterior hard palate and the apex towards the pre-maxillary anterior zone, obtained the lowest TRE value [32]. All these studies, however, were performed on phantoms, and different factors can interfere with the reliability of the procedure in relation to the surgical real condition, such as the different bone densities along the ZIs pathways, the presence of saliva or blood, the deformations of the surgical drills, and so on, called non-systematic errors [8]. In fact, the main drawback of this protocol consists of requiring a dedicated surgical approach to insert the screws in an atrophic bone, with risk of FM displacement during the next open flap surgeries. 

Newer DN systems, such as the one used in this case report, eliminated the necessity of bone anchored screws as FMs. Lopes et al. described a case of one ZI placement where the presence of three fixtures inserted in a previous rehabilitation allowed the operator to anchor a markers plate directly to the maxilla. The authors concluded that a simpler fiducial markers protocol could make the surgery faster and less complicated, considering that the screws insertion is time consuming and could condition the implant positioning [20].

The herein employed navigation system takes advantage from the placement of one mini-implant: this anchors the frame to the pre-maxilla, carrying the marker plate with three FMs and the patient reference tool [10,27]. Nevertheless, it must be noted that it is not simple to insert the mini-implant in an atrophic pre-maxilla: an incorrect mini-implant placement can cause interference of the marker plate with the handpiece, and a dislocation of the marker plate and the reference tool in case of loss of stability. 

It is useful that the provisional implant for maxilla anchoring, put in at least one month before surgery, can be used to support the provisional prosthesis in case of delayed prosthetic loading. The removal of this implant is not compulsory.

The major drawbacks of DN surgery are the economic costs and the long learning curve for a novel variety of ergonomics. This second aspect is particularly important due to the difficulty of steadily using the handpiece, since the operator is not able to simultaneously examine the computer screen and the surgical field. Augmented reality technology is intended to overcome this problem with the use of special glasses superimposing more imaging information to the vision of the surgeon [34]. Another problem related to ergonomics is the necessity to have an expert team dedicated to the set-up of the technological apparatus according the operational requirements, preserving the sterility of the surgical field. Three-dimensional hand gesture recognition based on a depth camera is going to attract increasing interest as an efficient method of touch-free interface [35]. 

In conclusion, the exposed minimally invasive characteristics should encourage the use of dynamic navigation systems with intraoral reference anchorages for the placement of zygomatic. Nevertheless, it needs to be underlined that this is a one-case report and that the described technique must be further investigated to validate and determine the correct position of the zygomatic implant in a cohort of patients.
